# Conventional Cigarette and E-Cigarette Smoking among School Personnel in Shanghai, China: Prevalence and Determinants

**DOI:** 10.3390/ijerph16173197

**Published:** 2019-09-01

**Authors:** Jingfen Zhu, Fanghui Shi, Gang Xu, Na Li, Jiahui Li, Yaping He, Jinming Yu

**Affiliations:** 1 School of Public Health, Fudan University, 130 Dongan Road, Shanghai 200032, China; 2 School of Public Health, Shanghai Jiao Tong University, 227 South Chongqing Road, Shanghai 200025, China

**Keywords:** conventional cigarette, electronic cigarette, school personnel, prevalence, determinants

## Abstract

Smoking cigarettes and e-cigarettes is widely popular among Chinese students. Considering that school personnel are considered role models in the student community, we investigated the prevalence and determinants of such behavior among high school personnel in China so as to provide references for future related intervention measures. We used a stratified cluster sampling design on a total number of 3311 school employees recruited from 33 representative schools. Complex sampling analysis and logistic regressions were used for univariate and multivariate analyses. Among 3194 participants that met the study criteria, 7.4% were cigarette users, and 3.6% e-cigarette consumers. For conventional cigarette smokers, certain characteristics, such as being older and male, attaining less education, and having been exposed to secondhand smoke, were associated with heavier smoking. Nevertheless, e-cigarette users were predominantly male and of younger age. Those who understood the hazards of conventional cigarette smoking had less inclination to smoke but were at a higher risk of e-cigarette use. Our analysis suggests that it is necessary to target different populations for monitoring and controlling conventional cigarette smoking and e-cigarette use respectively among school personnel. In addition, China is in need of more relevant and strict anti-smoking regulations.

## 1. Introduction

The prevalence of adolescent smoking in China has dramatically increased since the 1990s [1]. According to the 2014 China Youth Tobacco Report, nearly 6.4% of middle school students are smoking conventional cigarettes, and 1.2% have tried e-cigarettes [2]. Once adolescents start to smoke, they are at high risk of becoming regular smokers. In addition, the earlier one begins to smoke, the higher the risk of addiction later in life [3]. School personnel have been considered an important factor associated with student smoking behaviors [4]. School teachers and administrators have a clear illustrative effect on the students [4,5]. Although the Ministry of Education and the Ministry of Health in China issued “Opinions on Further Strengthening School Control of Tobacco Control” in 2010 to prevent school personnel from smoking around students, related survey results showed that nearly 13.3% of students reported seeing teachers smoking at school. Furthermore, this policy still does not include e-cigarette-related regulations, which suggests that the smoke-free school policy needs to be further strengthened. Bearing in mind that school personnel are considered role models in the student community, there is an urgent need for research on conventional cigarette and e-cigarette smoking among this population.

According to the 2015 China Adults Tobacco Survey [6], 22.1% of school personal smoke cigarettes (2.4% practice e-cigarette smoking), which is slightly lower than the national average. However, considering the special influence the school personnel can have on students, they should be paid more attention to. The health effects of conventional cigarette smoking are well established; it has been estimated that half of the smokers will die prematurely because of their smoking habit [7]. Nevertheless, the health effects of e-cigarettes, battery-powered devices that produce an experience similar to that of smoking a conventional cigarette, still remain controversial and poorly characterized [8,9]. Existing findings [10,11] suggest that among non-heavy smokers, e-cigarette use is positively associated with quantity and frequency of conventional cigarette smoking, contradicting the hypothesis that e-cigarette use reduces smoking.

So far, several surveys [12,13] have been conducted on conventional cigarette smoking behavior among the school personnel, generating numerous risk factors, including socioeconomic status, cigarette price, taxes, media and advertising exposure, lack of social support, and so on [14]. Additionally, some studies have investigated the effects of school personnel smoking behavior on the rate of adolescent smoking. Piontek et al. [15], for example, have reported a positive association between teacher conventional cigarette smoking on school grounds and student smoking behavior. Nikaj and colleagues [16] stated that smoking among teachers and non-teaching staff is associated with higher average cigarette consumption among student smokers; and younger school personnel appeared to be more influential in determining behaviors among youths. These findings imply that there also might be a connection between the use of e-cigarettes among school personnel and use of e-cigarettes among students.

Past studies [8,17] have demonstrated that exposure to e-cigarette advertising and lacking awareness of potential harm related to smoking are associated with a higher risk of use. Furthermore, young male Caucasian adults with higher educational levels are also more likely to be e-cigarette users. Although many studies have examined cigarette smoking habits among the general population [1,18,19,20], only a few [4,21] have been conducted in the population of school personnel. Additionally, compared with the general population, there are some special school personnel-related characteristics, such as school type, job role, and the enforcement of tobacco control rules at schools. Consequently, more research is needed to monitor the use of cigarettes among school personnel and investigate the related factors leading to a high rate of cigarette use, especially for e-cigarettes.

Up till now, besides modest data on the prevalence of e-cigarette use among adults that were gathered during the 2015 China Adults Tobacco Survey, only few population-based studies have estimated e-cigarette use among Chinese adults [2,6]. Even fewer studies have simultaneously explored conventional cigarette and e-cigarette use within the same population of school personnel. The present study aimed to estimate the use of conventional cigarettes and e-cigarettes among school personnel in China and examine their association with sociodemographic characteristics and other possible variables. This approach generates helpful information that can be used to control both kinds of cigarette smoking among school personnel, which in turn could help prevent and reduce conventional cigarette smoking and e-cigarette use among students. In addition, considering the existing limitations and lack of current school policies in China, this work aimed to emphasize the urgency of setting up and improving relevant regulations.

## 2. Materials and Methods

### 2.1. Data Sources

In the present study, we used a stratified cluster sampling design to select 33 schools from four districts (Huangpu District, Minhang District, Jiading District, and Putuo District,) in Shanghai from September 2017 to January 2018. All personnel working at the selected schools were chosen as participants in the study. A total of 3311 questionnaires were filled out, among which 3194 were valid. This survey used a self-administered, anonymous data collection procedure. The questionnaire was based on the Global School Personnel Survey (GSPS) [22] of the World Health Organization (WHO) and “questionnaires for monitoring smoking-related behaviors of the key population”, developed by the Chinese Center for Disease Control and Prevention; the questionnaires were delivered to participants through Wenjuanxing (an online survey platform https://www.wjx.cn/).

This study was approved by the Ethics Committee of the Shanghai Jiao Tong University School of Public Health (SJUPN-201703).

### 2.2. Measures

#### 2.2.1. Sociodemographic Characteristics

The sociodemographic information [22] included age (<40/≥40), sex (male/female), educational attainment (college or below/bachelor or postgraduate), and job position (teacher/non-teaching staff).

#### 2.2.2. Smoking Status and E-Cigarette Use

According to the Global School Personnel Survey (GSPS) [22], cigarette smoking status can include current smokers (participants who smoke cigarettes either daily or occasionally at the moment of the survey), former smokers (participants who do not smoke cigarettes at the time of the survey, but have smoked cigarettes in the past), and never-smokers (participants who have never smoked cigarettes). Thus, data on smoking status were obtained using the question: “Have you ever smoked conventional cigarettes?” [23]. Participants who selected from the following three answers: “No, I have never smoked cigarettes”, “Yes, and I still smoke cigarettes”, or “I smoked cigarettes in the past, but I don’t smoke any more” were defined as “never smokers”, “current smokers”, or “former smokers”, respectively.

Smoking e-cigarettes [24] was assessed using the following question: “Have you ever tried electronic cigarettes?”. Responses of “regularly” or “occasionally” were defined as “having tried an e-cigarette”. Those who chose “no” were defined as “never having tried an e-cigarette.”

#### 2.2.3. Secondhand Smoke Exposure and E-Cigarette Exposure

Participants [22] who reported not being around anyone smoking conventional cigarettes at home or public places over the past 7 days were defined as “not exposed to secondhand smoke (SHS)”. Other participants were defined as “exposed to SHS”. Respondents [25,26] who stated that they did not see anyone using e-cigarettes over the past 30 days were defined as “not exposed to e-cigarettes”. In addition, other respondents were categorized as “exposed to e-cigarettes”.

#### 2.2.4. Knowledge of Harmfulness of Conventional Cigarettes and Attitude toward Conventional Cigarettes

One multiple-choice question, “Which of the following health problems are related to smoking and secondhand smoke?”, was used to assess participants’ knowledge of the harmfulness of conventional cigarettes. The response options to both questions were six related diseases that could be caused by cigarette smoking or secondhand smoke. The total number of choices the participant chose in these two questions was calculated as the final score. The participants were then re-coded into two categories (0–7: low score; 8–12: high score) by median. Attitudes toward conventional cigarettes were assessed with the following statements: “Cigarettes with low tar and low nicotine are less harmful to the body”; “Passive smoking has little effect on health”; “Quitting smoking after short-term smoking has no impact on health”; and “Smoking addiction is a chronic disease”. Answers included: “agree”, “not sure”, and “do not agree”. If the participant chose “agree” for any of the former three questions or chose “do not agree” for the fourth question, we thought he or she had a positive attitude toward conventional cigarettes.

#### 2.2.5. Attitude toward E-Cigarettes

Attitudes toward e-cigarettes were assessed with the following statements [27,28]: “E-cigarettes are less harmful than conventional cigarettes,”, “E-cigarettes can help with smoking cessation,”, “E-cigarettes are more fashionable than conventional cigarettes”, and “E-cigarettes are not addictive”. Similar with the assessment of attitude toward conventional cigarettes, school personnel who chose “agree” for any of these questions were categorized as “having positive attitude toward e-cigarettes”, while other answers were categorized as having a negative attitude.

#### 2.2.6. Other Variables

The question, “Do you think your school has a rule that forbids school personnel from smoking?” was used to assess the enforcement of tobacco control regulation at school. The four possible responses were as follows: “There is no rule”, “Yes, but the rule isn’t enforced”, “Yes, the rule is sometimes enforced”, and “yes, the rule is strictly enforced”. Since the majority of school personnel thought their school’s tobacco rule was strictly enforced, we computed the percentage of school personnel who perceived the rule against tobacco as strictly enforced for each school. By the median (80%) percentage of the perceived strictly enforced anti-smoking rule, we then classified schools into strict and non-strict enforcement of anti-smoking rules. In addition, junior high school, senior high school, and vocational school personnel participated.

### 2.3. Data Analysis

The complex sampling analysis was used for the data considering stratification, clusters, and weights to represent the entire school personnel in Shanghai. All variables were presented as weighted percentages and respective 95% confidence intervals (CIs). Crude and adjusted odds ratios, with 95% CIs, were calculated for variables in the binary logistic regression analysis to identify the determinants of both conventional cigarette and e-cigarette use. A threshold for statistical significance was set at a two-tailed *p* < 0.05 level. All data were analyzed using SPSS, version 22.0.

## 3. Results

Table 1 shows the distribution of characteristics and smoking-related factors among school personnel. About half of the target population were ≥40 years old and 73.3% (95% CI: 71.8–74.8%) were female; 91.6% (95% CI: 90.6–92.5%) of the participants had a bachelor’s degree or higher educational attainment and 80.6% (95% CI: 79.2–81.9%) were teachers. Current conventional cigarette smokers and e-cigarette users comprised 3.8% (95% CI: 3.2–4.5%) and 3.6% (95% CI: 3.0–4.3%) of school personnel, respectively.

As shown in Table 2 and Table 3, 0.5% (95% CI: 0.3–0.8%) of the females were current smokers, whereas 0.9% (95% CI: 0.6–1.4%) of them were e-cigarette users. Higher rates of current smokers of conventional cigarettes were found among older participants (10.8%, 95% CI: 9.4–12.3%), males (26.4%, 95% CI: 23.6–29.4%), and individuals with lower educational attainment (26.8%, 95% CI: 22.2–32%). In addition, compared with teachers (5.4%, 95%CI: 4.6–6.3%), the prevalence of conventional cigarette use was higher among the non-teaching staff (15.6%, 95% CI: 13.1–18.5%) (Table 2). Similarly, a higher rate of those having used e-cigarettes was also found among males (11.0%, 95% CI: 9.1–13.3%), non-teaching staff (6.0%, 95% CI: 4.5–8.1%), and individuals with lower educational attainment (7.8%, 95% CI: 5.4–11.3%). E-cigarette use was somewhat higher among younger personnel (3.8%, 95% CI: 2.9–4.8%) compared to those over 40 years of age (3.5%, 95% CI: 2.7–4.5%), which was opposite to conventional cigarette use. Furthermore, personnel from vocational schools had higher use rates of both conventional cigarettes (14.4%, 95% CI: 12.1–16.9%) and e-cigarettes (5.9%, 95% CI: 4.5–7.8%).

Table 2 and Table 3 show the influencing factors of conventional and electronic cigarette use. Except for sociodemographic characteristics, exposure to SHS was the strongest predictor for current smokers (odds ratio (OR) = 2.24, 95% CI: 1.55–3.23), followed by a positive attitude toward conventional cigarettes (OR = 1.82, 95% CI: 1.32–2.51). The school’s rules on the enforcement and awareness of the hazards of conventional cigarette smoking resulted as independent, robust predictors in the univariate analysis according to the crude OR, although was not significant in the multivariate logistic regression. Compared to schools with a strict tobacco ban (5.6%, 95% CI: 4.5–7.0%) and personnel with high levels of awareness (5.6%, 95% CI: 4.6–6.8%), the prevalence of current cigarette smoking was higher in schools without a strict ban (8.8%, 95% CI: 7.7–10.2%) and among those with low awareness levels (9.1%, 95% CI: 7.8–10.6%). However, among e-cigarette users, besides sociodemographic characteristics, current cigarette smoking (OR = 5.85, 95% CI: 3.52, 9.75) was the strongest predictor of using e-cigarettes, followed by being exposed to e-cigarettes (OR = 3.2, 95% CI: 2.05, 4.98), having positive attitudes toward e-cigarettes (OR = 2.42, 95% CI: 1.58, 3.72), and attaining a high score of awareness on hazards related to conventional cigarette smoking (OR = 1.53, 95% CI: 1.01, 2.35).

Stratified analysis by gender showed that the influencing factors on traditional tobacco and e-cigarette use by school personnel of different genders were consistent with overall results mentioned above. However, a high score of conventional cigarette hazards (OR = 0.70, 95% CI: 0.53, 0.98) and ever being exposed to SHS (OR = 2.27, 95%CI: 1.56, 3.29) were only significantly correlated with the possibility of conventional cigarette smoking among males. A high score of conventional cigarette hazards (OR = 2.07, 95% CI: 1.26, 2.38) and positive attitudes toward e-cigarettes (OR = 2.59, 95% CI: 1.58, 4.24) were more likely to predict e-cigarette using among male school personnel, but not among female personnel. The results are shown in Appendix A Table A1 and Table A2.

## 4. Discussion

To the best of our knowledge, this is the first study that provided evidence on the prevalence and determinants of both conventional cigarette smoking and e-cigarette use among school personnel in China. Our results revealed that male non-teaching personnel from vocational schools were more likely to smoke conventional cigarettes and use e-cigarettes. However, contrary to conventional cigarettes users, those who used e-cigarettes were more likely to be of younger age. We also found that exposure to e-cigarettes and positive attitudes toward e-cigarettes increased the risk of using them, as well as using the conventional tobacco.

China is the largest tobacco consumer in the world, with a smoking rate of 27.7% among the adult population. Nevertheless, the prevalence of e-cigarette use among adults is much lower in China (3.1%) compared to the United States (13.5%) [8] and the European Union (11.6%) [19]. Our results showed that only 3.6% of the target population used e-cigarettes, which was higher than the average level of e-cigarettes use among all teachers across China (2.4%). Since e-cigarettes came into public view, their use has considerably increased across the world [29]. School personnel, as a special group whose behavior has a profound impact on the students, need to be further monitored for both conventional cigarette smoking behavior and e-cigarette use.

The propensity to use e-cigarettes is influenced by multiple factors [8,17]. Similar with most surveys [8,19], males in this current study had a higher prevalence of either conventional cigarette smoke or e-cigarette use than females. However, our results also showed that the conventional cigarette and e-cigarette smoking rates among males were 53 times and 12 times, respectively, that of females, which indicated a smaller gender difference in e-cigarette use compared to conventional cigarette use in China. Due to Chinese conventional cultural and the professional requirements of teachers, the smoking rate of conventional cigarettes among female teachers has always been low in China. Early in the year 2003, WHO reported that a gender gap in smoking rates was narrowing and the use of tobacco products was on the rise in the female population, especially among females in developing countries [30]. This may be because tobacco companies have started advertising tobacco products especially for women by associating smoking with the ideas of independence, body image, romance, etc. Females are also more likely to report preference for e-cigarettes because of their social impact, sweet flavor, and fashionable appearance [31]. Taken together, the higher use of e-cigarettes compared to conventional cigarettes among females reinforces the importance of tobacco control programs targeting the use of e-cigarettes among women. Considering that women are more likely to start using e-cigarettes (influenced by environmental stimuli) [31], more effective measures should be taken to control the prevalence of e-cigarettes among females.

Consistent with most of the previous studies [32], our results showed that younger adults were less likely to be conventional cigarette smokers and more likely to use e-cigarettes. It has been suggested that the e-cigarette industry targets young people, and a large number of advertisements are produced to attract them, which could help explain why younger personnel were more likely to use e-cigarettes [32]. Although a lot of studies have suggested that the use of e-cigarettes is higher among people with higher education attainment [8,9], our results showed no significant difference. This may be due to the fact that the subjects in this study were school educators whose educational level is often higher compared to the general population. Moreover, our results also showed that the current status of smoking conventional cigarettes and using e-cigarettes was twice to three times as high among non-teaching staff compared to teachers. Considering that different subgroups of school personnel were at higher risk of smoking conventional cigarettes or e-cigarettes, when targeting the population for control-related training or activities against the use of either kind of cigarettes, different aspects should be taken into consideration.

Apart from sociodemographic characteristics, participants’ attitudes toward e-cigarettes and conventional cigarettes were associated with smoking e-cigarettes or conventional cigarettes. It is widely accepted that a positive attitude toward conventional cigarettes leads to higher risk of using cigarettes. Studies also suggest that a positive attitude toward e-cigarettes is associated with a higher risk of using e-cigarettes [33]. Thus, it is necessary to educate the target groups on the harmfulness of the conventional cigarettes and e-cigarettes [34]. All the relevant information could be delivered through training and pertinent activities, which would enable school personnel to adopt a correct concept on conventional cigarettes and electronic cigarettes, eventually reducing the prevalence of both kinds of cigarettes. However, further studies are warranted to investigate the effective training content and its long-term impact on smoking behavior. Furthermore, many surveys have stated that students’ smoking attitude and behavior are greatly influenced by their teachers. However, most of the school personnel in China are not professionally trained in tobacco control skills for students [3] and some currently available training offered by schools only deals with the hazards of smoking. Thus, more efforts are needed to cultivate a more professional attitude in school personnel with respect to smoking, which could finally contribute to the reduction of smoking behavior among students. Additionally, because of the large population of students in China, it is necessary to search for cost-effective strategies to improve health education, such as making full use of new media.

The results of the current study showed that the exposure to SHS affected the use of conventional cigarettes, especially among the male population. Likewise, being exposed to electronic cigarettes also affected the use of e-cigarettes among school personnel. Although being exposed to SHS was not a risk factor for the use of e-cigarettes (OR = 1.04, *p* > 0.05), the e-cigarette use rate was still 1.6 times higher in the SHS exposure group compared with the non-exposure group. In China, the SHS exposure rate was on the decline; however, there were still 17.2% students who reported being exposed to SHS at school [6]. Up till now, besides some local level regulations, there are still no national smoke-free laws, which are the most effective way to protect people from SHS. According to a survey conducted in China in 2015 [6], the existing local smoking bans had a limited impact on preventing smoking in public places. In addition, almost none of the existing anti-smoking regulations in China limit the use of e-cigarettes. Given China’s high conventional tobacco use rate and rapidly increasing usage of e-cigarettes, it is necessary to enact and implement comprehensive national smoke-free laws in China.

Worldwide, school tobacco policies (STPs) limit tobacco use in schools by defining whether or where students and school personnel are allowed to smoke and by defining the penalties for those caught violating the smoking rules. A study in China reported that [35] when the bans are comprehensive and strictly enforced, a decreased smoking rate is more likely to occur at the school level. In June 2010, China issued a nationwide “Tobacco-Free School Policy”, which implied a total ban on smoking on campus, but it is still unclear to what extent it was implemented [36]. Although this rule explicitly prohibits faculty from smoking at school, a national survey conducted in 2014 reported that 54.5% of the students in China saw their teachers smoking on campus over the past 30 days [2]. Additionally, in China, the existing school tobacco control policy and related tobacco control education do not involve the use of electronic cigarettes. Therefore, the education sector should not only strengthen the implementation of smoke-free school policy, but also add restrictions to the use of electronic cigarettes at school.

The current smoking status is strongly correlated with e-cigarette use, which has been verified across different countries, including Canada [37], the United States [38], the EU countries [24], Serbia [29], etc. This is probably because cigarettes smokers are likely to use e-cigarettes as an aid to quit smoking or reduce smoking, thus treating e-cigarettes as a substitute for conventional cigarettes. Some studies [39] also argue that e-cigarettes may encourage nonsmokers to start using them, which might foster nicotine dependence and serve as a gateway to smoking conventional cigarettes, particularly among adolescents. Our results revealed that current smoking of conventional cigarettes was the strongest determinant of e-cigarette use, increasing the risk by nearly five times. Therefore, controlling the use of conventional cigarettes and electronic cigarettes are both very important because they seem to interact with each other. As school personnel is the main group that is in close contact with students, monitoring and controlling the use of conventional cigarettes and e-cigarettes among the school personnel is the key for tobacco control among students.

Limitations of this study include the cross-sectional design, through which it was possible to assess only association but not causal relationships. This limitation might be addressed by further longitudinal investigations and experimental studies that would inform the causal relationship between the use of e-cigarette and conventional cigarette smoking. In addition, although self-reporting is widely used, it still may have resulted in misclassification bias in this study. Finally, the results of this study can only be extended to school personnel in first-tier cities, such as Shanghai, but not to other cities or rural areas. Despite these limitations, this is the first study that investigated the prevalence and correlated factors of conventional cigarette smoke and e-cigarette use among school personnel in China.

## 5. Conclusions

The present study revealed that the use of conventional cigarettes among school personnel in Shanghai, China was lower than the national average level, while e-cigarette use was significantly higher than the national level. The prevalence of both kinds of cigarettes among school personnel was higher in male non-teaching staff at vocational schools and with low educational attainment. Personnel who were younger, aware of traditional tobacco hazards, exposed to e-cigarette vapor, had positive attitudes toward e-cigarettes, and were traditional tobacco users were more likely to use e-cigarettes. Given the significant impact of school personnel on tobacco use among students, it is important to strengthen tobacco control training among school staff, include the policy related to e-cigarette use, and create an effective social smoke-free environment.

## Figures and Tables

**Table 1:** Sample and population-weighted characteristics distribution among school personnel (N = 3194) in Shanghai, China.

	Un-Weighted Sample Size	Weighted	
		% (95% CI)	Number
School			
Junior high school	1719	58.2 (56.5–59.9)	50,394
Senior high school	681	27.0 (25.4–28.7)	23,411
Vocational school	794	14.7 (13.8–15.8)	12,750
Gender			
Male	887	26.7 (25.2–28.2)	23,082
Female	2307	73.3 (71.8–74.8)	63,474
Age group			
<40 y old	1479	47.2 (45.4–48.9)	40,841
≥40 y old	1715	52.8 (51.1–54.6)	45,714
Education			
Bachelor’s and above	2875	91.6 (90.6–92.5)	79,272
College and below	319	8.4 (7.5–9.4)	7283
Position			
Teachers	2521	80.6 (79.2–81.9)	69,775
Non-teaching staff	673	19.4 (18.1–20.8)	16,780
Smoking status			
Never	2805	88.8 (87.7–89.9)	76,884
Former	128	7.4 (6.5–8.3)	3299
Current	261	3.8 (3.2–4.5)	6373
E-cigarette use			
Never	3071	96.4 (95.7–97.0)	83,426
Ever	123	3.6 (3.0–4.3)	3130
Knowledge of conventional cigarette hazard			
Low score	1611	50.2 (48.5–52.0)	43,485
High score	1583	49.8 (48.0–51.5)	43,070
Attitude toward conventional cigarette			
Negative	1799	57.2 (55.5–59.0)	49,530
Positive	1395	42.8 (41.0–44.5)	37,026
SHS exposure			
No exposure	1116	35.4 (33.8–37.2)	30,681
Exposure	2078	64.6 (62.8–66.2)	55,875
School rule enforcement			
Strict	1298	45.3 (43.5–47.1)	39,206
Not strict	1896	54.7 (52.9–56.5)	47,349
Attitude toward e-cigarettes			
Negative	1942	61.2 (59.5–62.9)	52,976
Positive	1252	38.8 (37.1–40.5)	33,579
E-cigarette exposure			
No exposure	2725	85.9 (84.7–87.1)	74,362
Exposure	469	14.1 (12.9–15.3)	12,193

Note: Confidence interval (CI); secondhand smoke (SHS).

**Table 2:** Current conventional cigarette use and related influencing factors among school personnel in Shanghai, China.

	Weighted % (95% CI) ^a^	Crude OR (95% CI)	Adjusted OR (95% CI)
School			
Junior high school	5.9 (4.9–7.1)	ref = 1	ref = 1
Senior high school	6.8 (5.1–8.9)	1.16 (0.81–1.66)	0.94 (0.61–1.46)
Vocational school	14.4 (12.1–16.9)	2.69 (2.03–3.55) ***	1.25 (0.80–1.94)
Gender			
Male	26.4 (23.6–29.4)	ref = 1	ref = 1
Female	0.5 (0.3–0.8)	0.01 (0.01–0.02) ***	0.02 (0.01–0.03) ***
Age group			
<40 y old	3.5 (2.7–4.6)	ref = 1	ref = 1
≥40 y old	10.8 (9.4–12.3)	3.31 (2.43–4.49) ***	2.30 (1.64–3.24) ***
Education			
Bachelor’s and above	5.6 (4.8–6.5)	ref = 1	ref = 1
College and below	26.8 (22.2–32.0)	6.20 (4.61–8.34) ***	2.72 (1.71–4.32) ***
Position			
Teachers	5.4 (4.6–6.3)	ref = 1	ref = 1
Non-teaching staff	15.6 (13.1–18.5)	3.25 (2.49–4.62) ***	1.28 (0.86–1.92)
Knowledge of conventional cigarette hazard			
Low score	9.1 (7.8–10.6)	ref = 1	ref = 1
High score	5.6 (4.6–6.8)	0.59 (0.45–0.77) ***	0.74 (0.53–1.02)
Attitude toward conventional cigarette			
Negative	4.8 (3.9–5.9)	ref = 1	ref = 1
Positive	10.8 (9.3–12.5)	2.41 (1.84–3.17) ***	1.82 (1.32–2.51) ***
SHS exposure			
No exposure	4.6 (3.6–6.0)	ref = 1	ref = 1
Exposure	8.9 (7.7–10.1)	2.00 (1.46–2.74) ***	2.24 (1.55–3.23) ***
School rule enforcement			
Strict	5.6 (4.5–7.0)	ref = 1	ref = 1
Not strict	8.8 (7.7–10.2)	1.64 (1.24–2.17) ***	1.22 (0.81–1.85)

Note: Confidence interval (CI); odds ratio (OR). ^a^ Prevalence of conventional cigarette current smokers. *** *p* < 0.001.

**Table 3:** Electronic cigarette use and related influencing factors among school personnel in Shanghai, China.

	Weighted % (95% CI) ^a^	Crude OR (95% CI)	Adjusted OR (95% CI)
School			
Junior high school	2.7 (2.1–3.6)	ref = 1	ref = 1
Senior high school	4.3 (3.0–6.0)	1.58 (0.99–2.52)	1.83 (1.06–3.17)
Vocational school	5.9 (4.5–7.8)	2.24 (1.49–3.36) ***	1.70 (0.97–2.98)
Gender			
Male	11.0 (9.1–13.3)	ref = 1	ref = 1
Female	0.9 (0.6–1.4)	0.08 (0.05–0.12) ***	0.14 (0.08–0.24) ***
Age group			
<40 y old	3.8 (2.9–4.8)	ref = 1	ref = 1
≥40 y old	3.5 (2.7–4.5)	0.92 (0.64–1.34)	0.47 (0.30–0.73) ***
Education			
Bachelor’s and above	3.2 (2.6–3.9)	ref = 1	ref = 1
College and below	7.8 (5.4–11.3)	2.55 (1.62–4.03) ***	1.21 (0.62–2.34)
Position			
Teachers	3.0 (2.4–3.8)	ref = 1	ref = 1
Non-teaching staff	6.0 (4.5–8.1)	2.05 (1.39–3.03) ***	1.46 (0.88–2.43)
Knowledge of conventional cigarette hazard			
Low score	3.7 (2.8–4.7)	ref = 1	ref = 1
High score	3.6 (2.8–4.6)	0.98 (0.68–1.42)	1.53 (1.01–2.35) *
Attitude toward conventional cigarette			
Negative	2.6 (1.9–3.4)	ref = 1	ref = 1
Positive	5.0 (4.0–6.3)	1.80 (1.36–1.90) ***	1.03 (0.67–1.57)
SHS exposure			
No exposure	2.6 (1.8–3.8)	ref = 1	ref = 1
Exposure	4.2 (3.4–5.1)	1.63 (1.05–2.50) *	1.04 (0.65–1.68)
School rule enforcement			
Strict	3.2 (2.4–4.3)	ref = 1	ref = 1
Not strict	3.9 (3.1–4.9)	1.23 (0.84–1.80)	0.78 (0.47–1.29)
Attitude toward e-cigarettes			
Negative	2.2 (1.7–3.0)	ref = 1	ref = 1
Positive	5.8 (4.6–7.2)	2.70 (1.84–3.96) ***	2.42 (1.58–3.72) ***
E-cigarette exposure			
No exposure	2.5 (2.0–3.2)	ref = 1	ref = 1
Exposure	10.4 (7.9–13.6)	4.53 (3.08–6.66) ***	3.20 (2.05–4.98) ***
Smoking status			
Never and former smoker	2.0 (1.6–2.6)	ref = 1	ref = 1
Current smoker	23.8 (18.9–29.6)	15.24 (10.29–22.56) ***	5.85 (3.52–9.75) ***

Note: Confidence interval (CI); odds ratio (OR). ^a^ Prevalence of e-cigarette users. * *p* < 0.05. *** *p* < 0.001.

## Appendix A

**Table A1:** Related influence factors of traditional cigarette use among school personnel stratified by gender.

	Male		Female	
	Ora (95%CI)	*p* Value	Ora (95%CI)	*p* Value
School				
Junior high school	ref = 1		ref = 1	
Senior high school	0.92 (0.29–1.43)	0.551	1.92 (0.17–21.73)	0.291
Vocational school	1.07 (0.68–1.69)	0.942	6.02 (1.27–28.56)	0.001
Age group				
<40 y old	ref = 1		ref = 1	
≥40 y old	2.11 (1.49–2.99)	<0.001	-	-
Education				
Bachelor’s and above	ref = 1		ref = 1	
College and below	2.4 (1.47–3.89)	<0.001	9.57 (4.77–19.21)	<0.001
Position				
Teachers	ref = 1		ref = 1	
Non-teaching staff	1.28 (0.84–1.96)	0.251	0.87 (0.38–1.96)	0.617
Knowledge of conventional cigarette hazard				
Low score	ref = 1		ref = 1	
High score	0.70 (0.53–0.98)	0.038	1.33 (0.46–3.87)	0.613
Attitude to conventional cigarette				
Negative	ref = 1		ref = 1	
Positive	1.66 (1.20–2.21)	0.002	6.72 (1.38–32.74)	0.017
SHS exposure				
No exposure	ref = 1		ref = 1	
Exposure	2.27 (1.56–3.29)	<0.001	1.75 (0.58–5.33)	0.288
School rule enforcement				
Strict	ref = 1		ref = 1	
Not strict	1.19 (0.78–1.84)	0.408	1.95 (0.19–20.49)	0.431

Note: Confidence interval (CI); adjusted odds ratio (Ora).

**Table A2:** Related influence factors of electronic cigarette use among school personnel stratified by gender.

	Male		Female	
	Ora (95%CI)	*p* Value	Ora (95%CI)	*p* Value
School				
Junior high school	ref = 1		ref = 1	
Senior high school	2.11 (1.12–3.95)	0.021	1.26 (0.40–3.96)	0.340
Vocational school	1.62 (0.84–3.12)	0.143	2.12 (0.59–7.55)	0.795
Age group				
<40 y old	ref = 1		ref = 1	
≥40 y old	0.49 (0.29–0.82)	0.007	0.37 (0.14–0.96)	0.041
Education				
Bachelor’s and above	ref = 1		ref = 1	
College and below	0.69 (0.33–1.43)	0.316	0.46 (0.12–2.08)	0.314
Position				
Teachers	ref = 1		ref = 1	
Non-teaching staff	1.85 (1.01–3.38)	0.047	0.47 (0.16–1.39)	0.175
Low score	ref = 1		ref = 1	
High score	2.07 (1.26–2.38)	0.004	0.63 (0.26–1.51)	0.301
Attitude to conventional cigarette				
Negative	ref = 1		ref = 1	
Positive	0.85 (0.52–1.39)	0.528	2.18 (0.88–5.38)	0.090
SHS exposure				
No exposure	ref = 1		ref = 1	
Exposure	1.06 (0.61–1.85)	0.839	1.17 (0.44–3.15)	0.748
School rule enforcement				
Strict	ref = 1		ref = 1	
Not strict	0.80 (0.44–1.45)	0.456	0.75 (0.25–2.23)	0.604
Attitude toward e-cigarette				
Negative	ref = 1		ref = 1	
Positive	2.59 (1.58–4.24)	<0.001	1.81 (0.75–4.37)	0.189
E-cigarette exposure				
No exposure	ref = 1		ref = 1	
Exposure	3.09 (1.87–5.10)	<0.001	4.33 (1.64–11.37)	0.003
Smoking status				
Never and former smoker	ref = 1		ref = 1	
Current smoker	6.44 (3.75–11.08)	<0.001	-	-

Note: Confidence interval (CI); adjusted odds ratio (Ora).
